# Multislice Analysis of Blood Flow Values in CT Perfusion Studies of Lung Cancer

**DOI:** 10.1155/2017/3236893

**Published:** 2017-01-10

**Authors:** Silvia Malavasi, Domenico Barone, Giampaolo Gavelli, Alessandro Bevilacqua

**Affiliations:** ^1^Department of Electrical, Electronic and Information Engineering (DEI), University of Bologna, Viale Risorgimento 2, 40136 Bologna, Italy; ^2^Advanced Research Center on Electronic Systems (ARCES), University of Bologna, Via Toffano 2/2, 40125 Bologna, Italy; ^3^IRCCS-Istituto Scientifico Romagnolo per lo Studio e la Cura dei Tumori (IRST), 47014 Meldola, Italy; ^4^Department of Computer Science and Engineering (DISI), University of Bologna, Viale Risorgimento 2, 40136 Bologna, Italy

## Abstract

*Objectives*. Tumour heterogeneity represents a key issue in CT perfusion (CTp), where all studies are usually based on global mean or median values of perfusion maps, often computed on whole tumour. We sought to determine whether, and to what extent, such global values can be representative of tumour heterogeneity, with respect to single slices, and could be used for therapy assessment.* Materials and Methods*. Twelve patients with one primary non-small cell lung cancer lesion were enrolled in this study, for a total amount of 26 CTp examinations and 118 slices. Mean and median blood flow (BF) values, calculated voxel-based, were computed on each slice and the whole tumour. To measure functional heterogeneity, entropy was calculated on BF values as well.* Results*. Most of the slices were not represented by the global BF values computed on the whole tumour. In addition, there are a number of lesions having equivalent global BF values, but they are composed of slices having very different heterogeneity distributions, that is, entropy values.* Conclusions*. Global mean/median BF values of the single slices separately should be considered for clinical assessment, only if interpreted through entropy computed on BF values. The numerical equivalence between global BF values of different lesions may correspond to different clinical status, thus inducing possible errors in choice of therapy when considering global values only.

## 1. Introduction

The introduction of antiangiogenic therapies aiming at preventing or regularizing the growth of vascularization in cancer tissues has aroused lively interest around promising imaging techniques capable of detecting vascular changes, thus providing fundamental information about treatments effectiveness before the appearance of morphological changes. In the last few years, computed tomography perfusion (CTp) has gained a large consensus among researchers, thanks to its capability of providing both high morphological resolution images and functional information about the investigated tissues [1]. This noninvasive and widely available methodology allows obtaining time concentration curves (TCCs) pertaining to a specific region of interest (ROI) by repeatedly acquiring the same portion of tissue during, and after, the intravenous injection of an iodinated contrast agent [2]. Through the analysis of TCCs, it is possible to achieve colorimetric maps representing values of perfusion parameters that can be used for multiple purposes such as monitoring vascular changes after the administration of antiangiogenic drugs [3], predicting tumour response [4, 5], or differentiating tumour subtypes [6].

Several sources of variability are known to affect the reliability of final perfusion parameters [7] that from time to time have also prompted the scientific community to argue about the effectiveness of CTp [1, 8]. Indeed, when investigating the possible clinical applications, tumour heterogeneity, representativeness of tumour regions, reliability of results, and reproducibility of CTp examinations represent different as well as interconnected issues that should be addressed as a whole. In fact,* heterogeneity* is an intrinsic characteristic of all tumours [9, 10], at several levels, ranging from genes to tissues [11], and this is also reflected in the hemodynamic behaviour, for instance, in areas of angiogenesis and necrosis [12].

As a consequence, which part of the tumour could be the most* representative* one for clinical assessments has been widely debated. Traditionally, the first CTp examinations were performed on one slice, due to technology limitation of data acquisition and processing apparatuses [13]. Afterwards, the improvement of technology has permitted working on the whole tumour [14], or groups of slices, as the central ones [15]. However, authors have still continued working on a single section only [16], chosen as the one representing the largest tumour diameter [17], or better incorporated the solid-appearing part of the target lesion [18], or else being in the middle scan position [19–21]. Nonetheless, several researchers choose the single tumour section based on visual considerations only [22], such as that having the best quality [23], or the widest area [24], jointly to the least variability [25].

Undoubtedly, the most important issue to make CTp entering the clinical practice is the possibility of achieving between-patient and among-patients standardization. To this purpose, the* reproducibility *of* reliable* results is an essential requirement, but it must be coupled to the clinical representativeness of numerical results. In the literature, it has been widely stated that by considering the whole tumour [26], or even group of slices [27], perfusion parameters may improve reproducibility and repeatability [28], against a single slice. That is, considering a wider “population” (i.e., more slices), averaging values helps achieving a “global” tumour behaviour. Using global mean or median values can also work for diagnosis purposes, where CTp has been used to discriminate between benign and malignant in different types of lung lesions, including pulmonary solitary nodules. For instance, the overall mean of pulmonary index, pulmonary and bronchial blood flow (BF), is computed on multiple slices in [29] and on three tumour sections chosen according to the axial, coronal, and sagittal planes, in [30]. The overall median of all the CTp parameters for the whole tumour is computed in [14], where the median was preferred over the mean operator to avoid outliers.

All the studies considered refer to global perfusion parameters, whether they are mean or median values, encompassing all the tumour characteristics. However, in this way, besides the uncertainty intrinsic to the CTp acquisition and processing procedure, global parameters also reduce the variability due to tumour heterogeneity. This is acknowledged by several authors, which recognize that global values only provide an overall measure of variability [31] and that “may not be optimal for tumour evaluation prior to treatment or therapy response evaluation” [32]. Nevertheless, very few attempts have been made to try assessing the capability of CTp parameters to evaluate the treatment response of patients with non-small cell lung cancer (NSCLC), but the lack of reproducibility could not confirm the results. For instance, the study in [33], dealing with CTp monitoring of antiangiogenic therapies in lung cancer, concludes that CTp can detect therapy-induced changes in perfusion, but the lack of reproducibility depletes these findings. Similar outcomes regarding the CTp capability of monitoring antiangiogenic therapies were reported in [4], even though, in this case, no reproducibility studies have been performed. On the other hand, more recently the authors in [24] could not find any correlation between CTp parameters and survival of patients treated with antiangiogenic therapies and chemotherapy. Also, they concluded that entropy only, computed on the Hounsfield units (HU), could be considered as an independent prognostic factor for overall survival (OS), this suggesting the importance of tumour heterogeneity in assessing tumour aggressiveness.

While it is widely agreed that considering tumour volumes give more information than using a single slice, usually single slices are merged together to provide global parameters. The aim of this work is to investigate the clinical representativeness of global perfusion values and to assess their capability to deal with tumour heterogeneity. To this purpose, an extensive analysis is carried out on a wide set of 118 tumour slices, and on corresponding whole tumours, referring to 12 baseline and 14 follow-up (FU) examinations. BF values for each voxel of the various tumour sections were calculated and showed through the use of colorimetric maps. Global mean and median BF values of each slice and of the whole tumour were then computed. In order to measure hemodynamic heterogeneity, entropy was computed on the BF values of each colorimetric map and of each lesion. The entropy is a well-known measure of the information content [34] and, to the best of our knowledge, this is the first time that the entropy is computed on BF maps. Therefore, in this work the entropy is also a measure of the representativeness of the information content conveyed by a BF map.

The first statistical analysis was carried out to exclude that the groups of single slices composing the respective whole tumours had the same mean or median value. Then, a second analysis was executed to assess whether some slices exist with the same mean or median global BF values as the whole tumour, so as to find out which perfusion pattern, at several levels, is represented by the global values of whole tumours. In addition, being each slice endowed with its own entropy value, it is also possible to assess the heterogeneity those selected slices own. To complete the analyses, a comparison between tumours with same global mean or median BF values was performed, so as to verify whether, and to what extent, a statistical equivalence of global perfusion values hints at similar perfusion patterns and heterogeneity features.

During data analysis, the results achieved for baseline and FU examinations are kept separate in order to allow detecting possible differences between untreated lesions, preserving their natural vascular structure, and lesions whose vascular network has been modified by the action of antiangiogenic treatments.

## 2. Materials and Methods

This retrospective study was approved by the Institutional Review Board. Twenty-two patients (fifteen men, seven women, mean age 64.7 years, range 42–81 years) with one primary NSCLC lesion underwent CTp. Patients over eighteen, with lesions having the longest axial diameter larger than 15 mm in at least three sections, were considered. Lesions whose boundaries could not be accurately identified, such as in case of highly inflamed tissues surrounding the tumour, were excluded from the study. Finally, twelve patients (nine men, three women, mean age 64.7 years, range 42–81 years) with a target lesion having mean longest axial diameter of 43.5 mm (range 25.3–75.2 mm) and a mean area of 1625 mm^2^ (range 433–1995 mm^2^) were enrolled. Five of them underwent at least one FU, for a total amount of 26 CTp examinations.

### 2.1. CTp Protocol and Perfusion Maps

Axial CTp examinations were carried out using a 256-slice CT system (Brilliance iCT, Philips Medical Systems, Best, The Netherlands). Patients were instructed for breath-hold and laid in the supine (feet first) position. An initial full-body, unenhanced and low-dose, CT scan was performed to identify the target lesion at the baseline condition. Then, 50 mL of intravenous bolus of contrast agent (Iomeron, Bracco, Milan, Italy) was administered to the patients at 5 mL/s. Five seconds later, a CTp scan of 25-second duration was performed at fixed tube voltage (80 kV), current (250 mA), and exposure (100 mAs). The protocol yielded 20 scans, centred on the target lesion, with a *z*-coverage of 55 mm (11 slices × 5 mm thickness, 0.4-second rotation time), and rearranged into 220 cine images (512 × 512 pixel, 350 mm × 350 mm, 5 mm slice spacing, and 1.25-second temporal resolution). For each slice, the longest axial diameter was computed, using a digital calliper. Due to the inclusion criteria, the number of slices where tumours are visible changes for each examination, but they are almost always visible for at least five slices. For this reason, the set of the five central slices was selected [27], achieving 118 slices (57 for baseline CTp and 61 for FUs) altogether. Hereinafter, the set of slices of each tumour is referred to as the “whole tumour.”

Two 25-year experienced radiologists analysed the whole sequences in cine-mode fashion and in agreement, for each examination, placed a circular region of interest (ROI) within the aorta to extract the arterial input and, for each slice, drew a ROI following the margin of tumours. Voxel-based BF values, expressed in mL/min/100 g, were computed for each slice, using an in-house algorithm developed in Matlab (MathWorks, Natick, MA), implementing the maximum slope method [35] and represented through the use of colorimetric maps. Misleading perfusion values computed on poorly representative TCCs (due to a bad fit) were excluded from the analysis according to what is reported in [16, 36], in order to obtain more reliable results. The corresponding voxels are displayed in the colour maps with the “pink” colour. Mean BF values representative of each slice (*μ*_*s*_) and of the whole lesion (*μ*_*w*_) were computed for each examination. Median values were also computed for each slice (*M*_*s*_) and the whole lesion (*M*_*w*_). The ranges (*r*) between minimum and maximum of *μ*_*s*_ and *M*_*s*_, *r*_*μ*_ and *r*_*M*_, respectively, were computed as a variability measure referred to as the whole volume.

### 2.2. Heterogeneity Analysis of BF Maps

The entropy is a measure often used in texture analysis, also applied to the oncologic field, for instance, to get a measure of texture irregularities [12]. Besides that, the entropy, computed on both nonenhanced and contrast-enhanced CT images, has been shown to correlate with the overall survival in patients with colorectal cancer [37] and gliomas [38] and with tumour staging [39] and overall survival [24] in patients with NSCLC. In this work, the entropy, *E*, was computed for the first time on the BF maps of the whole tumour (*E*_*w*_) and of each slice (*E*_*s*_), with the purpose to get a measure of the hemodynamic heterogeneity. The range *r*_*E*_ between minimum and maximum *E*_*w*_ values is also considered as a measure of the heterogeneity variability in the whole tumour. *E* measures are reported in arbitrary units (a.u.). More details are given in the Appendix.

**Figure 1:**
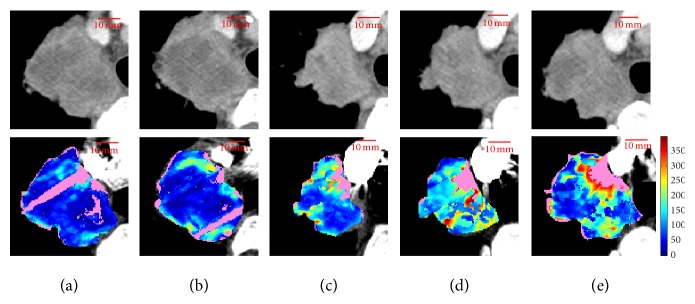
Original ROIs (HU, first row) and BF maps (second row) of all adjacent slices of lesion ID8, ordered by increasing *E*_*s*_ values: the slice at level 2 (*E*_*s*_ = 7.07) (a), level 1 (*E*_*s*_ = 7.43) (b), level 5 (*E*_*s*_ = 7.62) (c), level 4 (*E*_*s*_ = 7.92) (d), and level 3 (*E*_*s*_ = 8.10) (e). Values are reported in arbitrary units (a.u.).

Figure 1 reports an example of BF maps, referred to as lesion ID8, ordered from (a) to (e) according to increasing *E*_*s*_ values.

### 2.3. Statistical and Data Analysis

Statistical analysis was performed by using statistical software (R, version 3.2.1, The R Foundation for Statistical Computing). *p* values ≤0.05 were considered for statistical significance. Kendall-*τ* coefficient was used to assess any possible correlation between measurement errors and their magnitude: in case of concordance, data were log-transformed. Three groups of statistical tests were performed.

First, the one-way analysis of variance (ANOVA) was performed to check whether all slices (the “groups”) of the same tumour have the same mean value, that is, whether they can represent the same population, in terms of BF values. An analogous assessment was carried out for medians, through the Chi-squared test of independence. The second group consists in the two-tail *t*-test and the Wilcoxon rank sum test, which were utilized for three different purposes. In fact, they were applied to test, for each lesion, the difference of means and medians, respectively, between each slice and the whole tumour, with the purpose to check whether a slice exists which can represent the whole tumour (i.e., having the same global value). The same tests were also carried out to check for *μ*_*s*_ or *M*_*s*_ differences between couples of slices, whether they belonged to same tumour or different ones. Finally, they were employed to select which tumours have the same statistical *μ*_*w*_ or *M*_*w*_ values, to further compare their perfusion patterns (i.e., their *E*_*w*_). In fact, computing and using a global mean, or median, perfusion value for CTp studies implicitly means that sets of BF values (e.g., slices or whole tumours) with same *μ*_*s*_ (or *M*_*s*_) as *μ*_*w*_ (or *M*_*w*_) are clinically equivalent. The third group of tests is composed by the one-tail *t*-test only, which was performed to assess the differences between the means of *E*_*s*_ for baseline and FU examinations.

## 3. Results

The goal of this section is assessing the capability of global values, computed on the whole tumour, to represent the clinically relevant perfusion features of a tumour, assuming that the heterogeneity is among the most important ones [40]. To this purpose, we addressed tumours with different heterogeneities, referring to baseline and FUs examinations, by comparing those with the same global value. In addition, whole tumours and their composing slices with same global values were compared as well. In this section, we aim to check whether, and to what extent, numerical equivalence matches with clinical one.

As the first outcome, it is worth reporting that the hypotheses that means or medians of slices were all equivalent were rejected, for each examination. Actually, this finding was expected and suggests that the variability between slices is significantly greater than the variability within slices [41].

Tables 1 and 2 report the most significant measures (entropy, mean, and median) for all examinations, calculated on BF values of each slice and of the whole tumour. The range of measures is also reported. For the sake of brevity, in this section we just report a subset of the most interesting cases.

### 3.1. Baseline CTp

**Figure 2:**
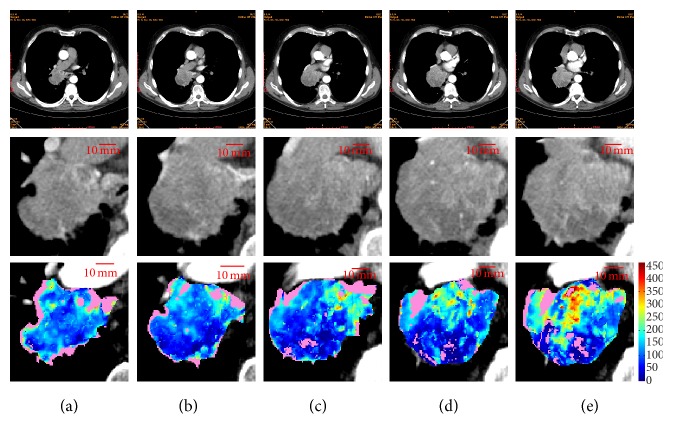
ID12: the whole scan (first row), HU (second row), and BF maps (third row) ordered from (a) to (e) according to the scan position (the third section is the central one). The BF maps are visualized using the same colour scale. By chance, they are also sorted according to their *E* value: *E*_*s*_ = 7.48 (a), *E*_*s*_ = 7.64 (b), *E*_*s*_ = 7.86 (c), *E*_*s*_ = 8.01 (d), *E*_*s*_ = 8.37 (e).

Table 1 resumes the most significant measures for the baseline CTp examinations. Statistical analysis shows that ten slices exist which have the same global BF as the respective whole tumour, seven times regarding mean values, and eight ones median values. Five times the whole tumour could be represented by the same slice detected by both *μ*_*w*_ and *M*_*w*_ values. *μ*_*w*_ and *M*_*w*_ values never selected the slice with maximum *E*_*s*_ and one time selected the slice with minimum *E*_*s*_ (ID12 and ID4, for mean and median, respectively).  Figure 2 reports the five slices of ID12, one of the most interesting lesions, where the average BF value of the whole tumour (*μ*_*w*_ = 125.0) corresponds to that of the first slice (*μ*_*s*_ = 124.5, Figure 2(a), last row). It is worth noting that this slice also retains the minimum *E*_*s*_ = 7.48, that is, the lowest heterogeneity. In fact, it shows quite a uniform, low, perfusion. On the contrary, the last slice (Figure 2(e)) shows a marked heterogeneity, the highest one (*E*_*s*_ = 8.37), having in its upper part a hyperperfused region (with BF values higher than 300), and a lower hypoperfused region with BF values nearly 40.

### 3.2. Follow-Ups CTp

**Figure 3:**
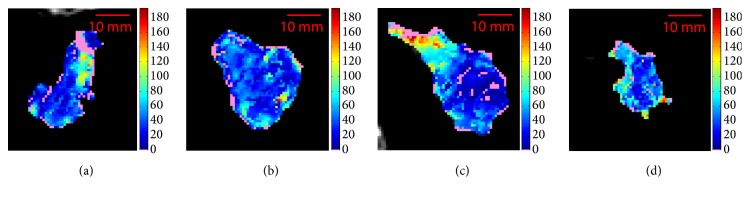
BF maps of the four consecutive slices of ID6-FU3 (1–4, from (a) to (d)). *μ*_*w*_ = 44.4, *M*_*w*_ = 37.8.

**Figure 4:**
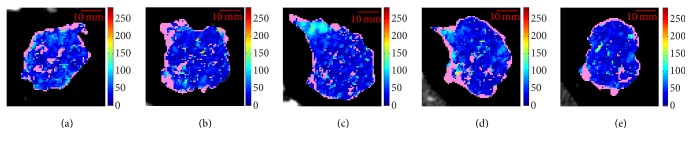
BF maps of the five consecutive slices of ID6-FU4 (1–5, from (a) to (e)). *μ*_*w*_ = 39.2, *M*_*w*_ = 33.2.

**Figure 5:**
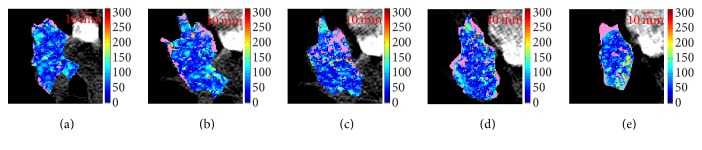
BF maps of the five consecutive slices of ID11-FU4 (1–5, from (a) to (e)). *μ*_*w*_ = 72.3, *M*_*w*_ = 63.3.

Table 2 resumes the most significant measures for the FU CTp examinations. Fifteen slices were representative of the whole tumour, thirteen of which regarding mean BF values, and eight pertaining to median values. Five times, mean and median global BF values identified the same slice. For lesions ID6-FU3 (Figure 3, *μ*_*w*_ = 44.4 and *M*_*w*_ = 37.8) and ID6-FU4 (Figure 4, *μ*_*w*_ = 39.2 and *M*_*w*_ = 33.2), the same slices (i.e., slice 3 for both) were those with maximum *E* (*E*_*s*_ = 6.73 and *E*_*s*_ = 6.49, respectively), probably due to these examinations being subsequent FUs of the same lesion. In addition, this is the only ID where mean and median select the highest *E*. As regards ID6-FU4, it shows limited BF ranges (*r*_*μ*_ = 7.3 and *r*_*M*_ = 8.0, among the lowest values of all examinations) and *μ*_*s*_ and *M*_*s*_ are substantially equivalent for the three central slices. This consideration regarding mean range also holds for ID6-FU3, where *r*_*μ*_ = 13.8 is a little higher, but still among the lowest ones. As for median, in ID6-FU3 it also selects slice 2 (*M*_*s*_ = 36.9) that has the lowest *E*_*s*_ = 6.26. On the other hand, in ID11-FU4 (Figure 5), *M*_*w*_ = 63.3 selects slice 1 (*M*_*s*_ = 64.3) which is the one with the lowest *E*_*s*_ = 7.05.

### 3.3. Baseline and FU CTp

**Figure 6:**
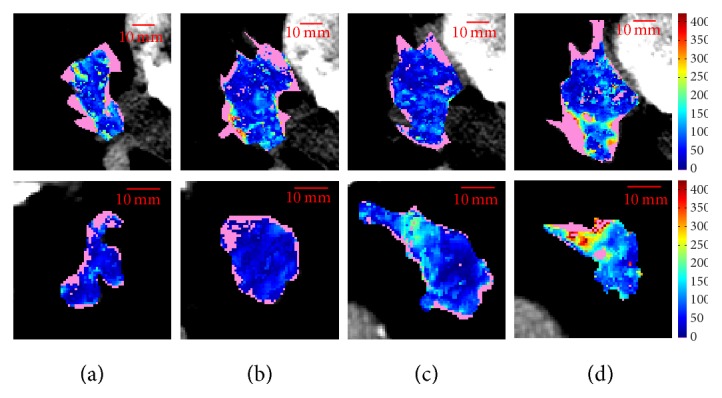
BF maps of the four consecutive slices (1–4, from (a) to (d)) composing lesions ID11, baseline (*μ*_*w*_ = 80.0, first row) and lesion ID6-FU1 (*μ*_*w*_ = 77.5, second row). *μ*_*w*_'s are statistically equivalent.

In this section, we extend the analysis over the whole dataset, by considering all the CTp examinations together. As regards the whole sets of slices, the most meaningful result is that on the whole 93 slices were not represented by the global BF values computed on the whole tumour. As for the sets of whole tumours, here we analyse the sets of slices referring to two couples of meaningful lesions.  Figure 6 shows the BF maps of the four consecutive slices (1–4, from (a) to (d)) of ID11 (*μ*_*w*_ = 80.0, first row) and ID6-FU1 (*μ*_*w*_ = 77.5, second row). Although these lesions have statistically equivalent *μ*_*w*_, the respective composing slices have a different heterogeneity distribution. In fact, the heterogeneity in all slices (except for slice 3) of ID11 is quite comparable, as it can be seen from *E*_*s*_ values of Table 1. On the contrary, slices 1 and 2 of ID6-FU1 (Figures 6(a) and 6(b), second row) are quite homogeneous and low-perfused, while slice 4 (Figure 6(d), second row) has the highest *μ*_*s*_ = 150.8 and *E*_*s*_ = 7.75. In addition, here the heterogeneity is made of local homogeneities, with a hyperperfused upper region and a hypoperfusion in the lower one.

**Figure 7:**
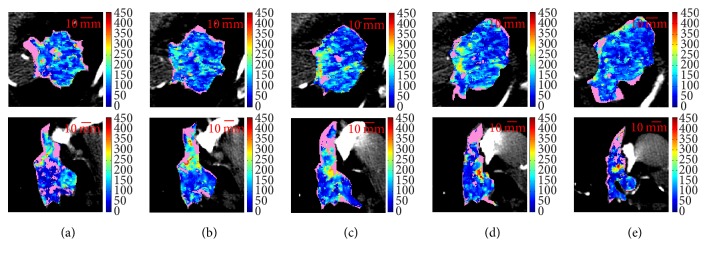
BF maps of the five consecutive slices (1–5, from (a) to (e)) composing lesions ID3, baseline (*μ*_*w*_ = 111.8, first row) and lesion ID2-FU1 (*μ*_*w*_ = 112.8, second row). *μ*_*w*_'s are statistically equivalent.

Similar comments can be done for ID3 and ID2-FU1, made of five slices each, whose BF maps are shown in Figure 7, first and second row, respectively. ID3 (*μ*_*w*_ = 111.8) shows a heterogeneity that keeps quite “homogeneous” within all slices (*r*_*E*_ = 0.23, the second lowest value), also in terms of mean (*r*_*μ*_ = 15.2) and median (*r*_*M*_ = 10.2) BF (among the lowest values), with all *μ*_*s*_ around *μ*_*w*_ = 111.8. On the other hand, the heterogeneity in ID2-FU1 (*μ*_*w*_ = 112.8) is made of well-defined hyper- and hypoperfused regions, mostly evident in the upper and lower part, respectively, of slices 2 and 3.

**Figure 8:**
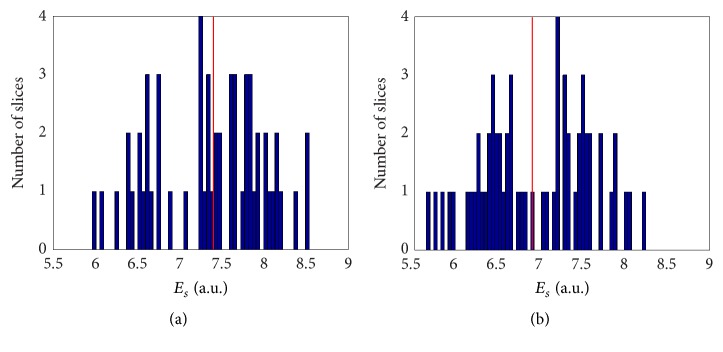
Histograms of entropies *E*_*s*_ of all slices for baseline (a) and FU (b) examinations. Mean values, highlighted by the vertical red lines, are 7.4 and 6.9, respectively.

Finally, we also analysed the distribution of all slice entropies *E*_*s*_ for baseline and FU examinations, separately. Related histograms are reported in Figures 8(a) and 8(b), respectively. Even at a glance, the histograms of baseline examinations appear shifted right with respect to the FU ones. In fact, for baselines mean and standard deviation are 7.4 and 0.75, respectively, while for FUs they are 6.9 and 0.64. Statistical tests confirm that the mean entropy of all slices is greater for baseline examinations (*p* values ≤10^−4^).

## 4. Discussion

Quantitative imaging has gained an increasing interest in these last years, as the need of personalized therapies progresses [42], deepening the knowledge of tumour's heterogeneity, the most important intrinsic properties of tumours. In particular, perfusion's heterogeneity is from decades a well-known characteristic of many tumours [40]. However, functional results obtained from CTp are still analysed using global statistical indexes, such as mean or median operators, that many times permit measurement reproducibility, while disregarding tumour heterogeneity [43]. Together with the uncertainty on reliability of voxel-based perfusion measurements, this represents one of the most relevant causes preventing the diffusion of CTp in clinical oncology, mainly to assess the outcome of therapies, such as antiangiogenic treatments.

In this work, we have deeply analysed the representativeness of global mean and median values, as far as the heterogeneity is concerned, starting from the assumption that computing and using a global mean, or median, perfusion value for clinical purposes means accepting that the characteristics of the tumour are represented by that value alone. Accordingly, this implies that sets of BF values (e.g., slices or whole tumours), with statistically equivalent mean or median values, are equivalently representative. The outcome of this work proves that these are numerical equivalences only, not clinical ones. In fact, we have discussed lesions with same global mean or median BF values, which showed a very different heterogeneity. In addition, we analysed tumour slices having the same global values as the whole tumour and we realized that, when those slices existed, for baseline examinations they were never those with the highest information content. Rather, it happened that in two examinations the whole tumour had mean and median BF values corresponding to the slice with the lowest heterogeneity (ID12 and ID4, respectively), while the remaining slices showed relevant clinical signs of different heterogeneities.

For FU examinations, there was only the wide, and highly necrotic, uniform lesion ID6 where mean and median selected the region with the highest *E*, in two subsequent FU examinations. In addition, results prove that using the median as a more robust estimator is not so effective as expected, since mean and median practically select the same slices. Rather, in ID6-FU3, the median operator selects the slices with maximum and minimum *E*_*s*_, at the same time. This behaviour has relevantly misleading properties, all the more so because median is considered a powerful outlier removal. Actually, this is true, if erroneous values lay in the extreme of values domain, but median cannot work in case that discriminating BF outliers strongly depends on spatial displacement and arrangement of BF values themselves.

## 5. Conclusion

Global perfusion values computed on the whole tumour cannot be appropriate for therapy assessment and cannot improve the reproducibility of heterogeneity, accordingly. In addition, we have shown that the global values computed on the whole tumour have a correspondence with parts of tumour (i.e., slices) that, just occasionally, could have either maximum or minimum entropy. In the remaining cases, they have not any correspondence with any real parts of the tumour and just represent a generic tumour BF averaged behaviour, which is far from representing its real clinical features. And perhaps, this happens much more times than expected. As far as single slices are concerned, although preserving more details, they may be not representative of the clinical status of the whole lesion and this could severely mislead clinical considerations.

The solution is not at hand, but the research carried out in this work suggests that the first step is being very prudent in considering the global (mean or median) BF values as useful indicators for therapy assessment. On the other hand, measuring heterogeneity is a key issue to achieve useful information to assess the effectiveness of antiangiogenic therapies that cannot be left out of consideration. This is confirmed by the comparison between the average BF entropy of all slices before (baseline) and after (FU) treatment, proving the effectiveness of treatments themselves, expectedly reducing the overall BF heterogeneity of tumours. We have also seen that the single slices of a tumour are widely varying from each other and can represent different BF heterogeneity patterns that, on the whole, could provide a radiologist with an overall view of the whole tumour. Indeed, using all the single slices of a tumour, endowed with global BF values and a BF heterogeneity measures, would represent a step forwards, useful to help radiologists to draw more reliable clinical considerations.

As concluding notes, we believe that improving the reliability of voxel-based perfusion values has to be coupled with the reproducibility assessment of heterogeneity measurements. To this purpose, a deeper application of the bioengineering and computer science techniques to CTp data processing, in a multidisciplinary team, will play a key role in the next future to help translation of CTp into clinics.

The achievements of this work could be also assessed using other perfusion parameters, such as the blood volume and the mean transit time. In addition, a study dedicated to validate the BF entropy as a surrogate biomarker for the overall survival is being carried out, based on the existing correlation between BF entropy and tumour grading.

## Figures and Tables

**Table 1:** Summary of measures for slices and whole tumour referred to baseline CTp examinations: entropy (*E*), mean (*μ*), and median (*M*) BF values, where the subscripts *s* and *w* stand for *slice* or *whole*, respectively. For the whole tumour, *r* of each slice-based measure is computed as well. Italicized and bold-italicized values point out minimum and maximum value of a given measure, respectively. Bold nonitalicized values highlight an equivalence between *μ*_*s*_ and *μ*_*w*_, or *M*_*s*_ and *M*_*w*_.

Baseline examinations									
Patient	Slice						Whole tumor		
	Measure	1	2	3	4	5	Measure	Value	*r*
ID1	*E*_*s*_	***7.27***	***7.27***	7.26	6.75	*6.61*	*E*_*w*_	7.14	0.66
	*μ*_*s*_	67.9	62.9	62.3	45.8	40.5	*μ*_*w*_	56.0	27.4
	*M*_*s*_	56.9	50.9	45.7	33.8	28.4	*M*_*w*_	41.4	28.5

ID2	*E*_*s*_	8.17	8.15	***8.22***	8.05	*7.91*	*E*_*w*_	8.20	0.31
	*μ*_*s*_	124.1	115.8	128.5	**118.2**	108.3	*μ*_*w*_	**120.1**	20.2
	*M*_*s*_	107.3	96.6	110.8	**99.8**	93.2	*M*_*w*_	**102.0**	17.6

ID3	*E*_*s*_	7.81	7.75	***7.89***	7.85	*7.67*	*E*_*w*_	7.87	0.23
	*μ*_*s*_	117.6	**111.3**	116.4	115.1	102.3	*μ*_*w*_	**111.8**	15.2
	*M*_*s*_	101.3	**103.1**	102.4	103.7	93.5	*M*_*w*_	**100.1**	10.2

ID4	*E*_*s*_	*7.26*	7.38	7.31	7.36	***7.44***	*E*_*w*_	7.39	0.18
	*μ*_*s*_	65.7	69.7	65.1	**68.1**	71.2	*μ*_*w*_	**68.0**	6.1
	*M*_*s*_	**54.9**	56.9	47.9	50.0	53.6	*M*_*w*_	**52.8**	9.0

ID5	*E*_*s*_	7.81	***7.84***	7.36	*6.60*	7.79	*E*_*w*_	7.70	1.23
	*μ*_*s*_	115.1	106.8	80.7	43.2	107.3	*μ*_*w*_	87.2	71.8
	*M*_*s*_	93.2	87.1	**67.4**	38.2	90.6	*M*_*w*_	**67.6**	55.0

ID6	*E*_*s*_	6.10	*5.96*	6.38	***6.62***	6.59	*E*_*w*_	6.54	0.66
	*μ*_*s*_	33.3	31.7	41.3	52.7	63.9	*μ*_*w*_	42.9	32.2
	*M*_*s*_	27.5	28.5	**34.5**	47.0	58.8	*M*_*w*_	**36.8**	31.3

ID7	*E*_*s*_	6.37	*6.27*	***6.53***	—	—	*E*_*w*_	6.67	0.26
	*μ*_*s*_	**46.2**	38.2	60.9	—	—	*μ*_*w*_	**47.9**	22.7
	*M*_*s*_	**38.6**	30.5	55.3	—	—	*M*_*w*_	**40.7**	24.8

ID8	*E*_*s*_	7.43	*7.07*	***8.10***	7.92	7.62	*E*_*w*_	7.95	1.03
	*μ*_*s*_	74.9	61.5	138.4	156.0	118.6	*μ*_*w*_	105.2	94.5
	*M*_*s*_	57.1	53.9	119.0	141.7	104.1	*M*_*w*_	85.3	87.8

ID9	*E*_*s*_	***8.54***	*7.59*	7.65	8.03	8.53	*E*_*w*_	8.24	0.95
	*μ*_*s*_	175.7	79.6	81.4	104.6	160.1	*μ*_*w*_	118.4	96.1
	*M*_*s*_	158.3	60.0	62.3	73.0	133.2	*M*_*w*_	88.9	98.3

ID10	*E*_*s*_	6.75	6.66	*6.42*	6.52	***6.76***	*E*_*w*_	6.66	0.34
	*μ*_*s*_	45.9	**44.2**	38.2	40.8	48.5	*μ*_*w*_	**43.4**	10.3
	*M*_*s*_	37.8	**37.3**	32.5	34.6	42.6	*M*_*w*_	**36.4**	10.1

ID11	*E*_*s*_	7.46	7.34	*6.89*	***7.63***	—	*E*_*w*_	7.50	0.74
	*μ*_*s*_	92.2	**80.7**	58.9	92.6	—	*μ*_*w*_	**80.0**	33.8
	*M*_*s*_	76.5	**61.4**	51.3	74.1	—	*M*_*w*_	**63.3**	25.2

ID12	*E*_*s*_	*7.48*	7.64	7.86	8.01	***8.37***	*E*_*w*_	8.07	0.89
	*μ*_*s*_	**124.5**	110.0	113.6	116.8	157.4	*μ*_*w*_	**125.0**	47.4
	*M*_*s*_	118.7	106.1	104.0	112.7	145.9	*M*_*w*_	116.0	41.9

**Table 2:** Summary of measures for slices and whole tumour referred to FU CTp examinations. Notations are the same as those in Table 1. Here, the FU number is also reported.

Follow-up examinations										
Patient	FU	Measure	Slice					Whole tumour		
			1	2	3	4	5	Measure	Value	*r*
ID2	1	*E*_*s*_	7.85	***8.06***	7.99	7.88	*7.72*	*E*_*w*_	8.08	0.34
		*μ*_*s*_	99.6	130.6	118.3	**111.4**	97.6	*μ*_*w*_	**112.8**	33.0
		*M*_*s*_	85.2	110.4	99.9	85.6	75.2	*M*_*w*_	92.4	35.2

ID6	1	*E*_*s*_	6.50	*6.26*	7.33	***7.70***	—	*E*_*w*_	7.50	1.44
		*μ*_*s*_	47.7	38.0	**76.8**	145.6	—	*μ*_*w*_	**77.5**	107.6
		*M*_*s*_	41.1	33.6	70.4	120.7	—	*M*_*w*_	60.1	87.1
	2	*E*_*s*_	6.63	*6.21*	6.40	***6.90***	—	*E*_*w*_	6.82	0.69
		*μ*_*s*_	58.1	42.2	45.8	80.0	—	*μ*_*w*_	53.5	37.8
		*M*_*s*_	49.2	38.6	32.5	71.4	—	*M*_*w*_	43.2	38.9
	3	*E*_*s*_	6.37	*6.26*	***6.73***	6.43	—	*E*_*w*_	6.67	0.47
		*μ*_*s*_	40.8	40.9	**45.7**	54.6	—	*μ*_*w*_	**44.4**	13.8
		*M*_*s*_	33.3	**36.9**	**36.3**	50.4	—	*M*_*w*_	**37.8**	17.1
	4	*E*_*s*_	6.42	6.31	***6.49***	6.42	*6.18*	*E*_*w*_	6.45	0.31
		*μ*_*s*_	42.7	**38.1**	**40.3**	**39.3**	35.3	*μ*_*w*_	**39.2**	7.3
		*M*_*s*_	38.6	31.8	**32.8**	**33.2**	30.6	*M*_*w*_	**33.2**	8.0
	5	*E*_*s*_	***7.47***	6.14	5.94	*5.64*	5.75	*E*_*w*_	6.50	1.84
		*μ*_*s*_	96.1	33.3	24.7	19.9	24.7	*μ*_*w*_	35.8	76.3
		*M*_*s*_	91.8	29.1	19.4	16.4	21.7	*M*_*w*_	24.7	75.4

ID7	1	*E*_*s*_	***7.57***	*6.57*	7.20	—	—	*E*_*w*_	7.64	1.00
		*μ*_*s*_	145.5	52.2	**97.7**	—	—	*μ*_*w*_	**95.5**	93.3
		*M*_*s*_	133.8	42.6	78.4	—	—	*M*_*w*_	78.4	91.2
	2	*E*_*s*_	*5.82*	***6.33***	5.90	—	—	*E*_*w*_	6.33	0.51
		*μ*_*s*_	34.1	48.8	58.0	—	—	*μ*_*w*_	45.0	23.8
		*M*_*s*_	32.2	42.8	56.7	—	—	*M*_*w*_	39.8	24.4

ID9	1	*E*_*s*_	***7.45***	*6.47*	6.60	6.50	6.58	*E*_*w*_	6.80	0.98
		*μ*_*s*_	80.8	36.9	**45.1**	38.7	41.6	*μ*_*w*_	**46.2**	43.8
		*M*_*s*_	67.6	31.4	39.6	34.1	36.2	*M*_*w*_	38.4	36.2

ID11	1	*E*_*s*_	7.04	6.83	*6.63*	***7.46***	—	*E*_*w*_	7.20	0.83
		*μ*_*s*_	71.4	54.7	50.4	108.4	—	*μ*_*w*_	66.9	58.0
		*M*_*s*_	60.7	47.3	44.6	96.5	—	*M*_*w*_	55.5	51.9
	2	*E*_*s*_	6.77	*6.66*	7.26	7.49	***7.53***	*E*_*w*_	7.40	0.87
		*μ*_*s*_	52.4	43.3	**67.7**	90.9	107.9	*μ*_*w*_	**70.2**	64.6
		*M*_*s*_	41.7	33.6	**55.6**	84.0	95.9	*M*_*w*_	**56.5**	62.3
	3	*E*_*s*_	7.20	*7.17*	7.30	***7.49***	—	*E*_*w*_	7.40	0.32
		*μ*_*s*_	74.8	73.2	**75.9**	90.5	—	*μ*_*w*_	**78.1**	17.3
		*M*_*s*_	70.4	66.2	**69.7**	81.0	—	*M*_*w*_	**71.0**	14.8
	4	*E*_*s*_	*7.05*	7.20	7.22	7.28	***7.32***	*E*_*w*_	7.29	0.27
		*μ*_*s*_	69.0	**72.9**	**70.9**	**71.5**	79.0	*μ*_*w*_	**72.3**	10.1
		*M*_*s*_	**64.3**	**64.1**	60.1	61.1	70.1	*M*_*w*_	**63.3**	10.0
	5	*E*_*s*_	7.57	*7.39*	7.52	7.87	***8.24***	*E*_*w*_	7.81	0.85
		*μ*_*s*_	80.9	71.7	76.7	99.9	144.7	*μ*_*w*_	90.1	73.0
		*M*_*s*_	60.7	55.2	61.3	74.2	123.2	*M*_*w*_	66.8	68.0

## Appendix

### Entropy

The Shannon entropy (or briefly, entropy) was introduced in the Information Theory in the late 1949 [34] as a useful tool able to quantify the information content. Since then, this feature has been widely applied in many fields, image analysis included. The entropy (*E*) is usually computed according to (A.1) [12]:

(A.1) $$\begin{array}{c} E=-\underset{i}{\sum }p\left(\;v_{i}\;\right)\log_{2}\;p\left(\;v_{i}\;\right), \end{array}$$

where *p*(*v*_*i*_) represents the frequency of the* i*th BF values inside the BF map.
